# Lack of Conventional Acinar Cells in Parotid Salivary Gland of Patient Taking an Anti-PD-L1 Immune Checkpoint Inhibitor

**DOI:** 10.3389/fonc.2020.00420

**Published:** 2020-04-02

**Authors:** Sarah Pringle, Bert van der Vegt, Xiaoyan Wang, Nico van Bakelen, T. Jeroen N. Hiltermann, Fred K. L. Spijkervet, Arjan Vissink, Frans G. M. Kroese, Hendrika Bootsma

**Affiliations:** ^1^Department of Rheumatology and Clinical Immunology, University of Groningen, University Medical Center Groningen, Groningen, Netherlands; ^2^Department of Pathology and Medical Biology, University of Groningen, University Medical Center Groningen, Groningen, Netherlands; ^3^Department of Oral and Maxillofacial Surgery, University of Groningen, University Medical Center Groningen, Groningen, Netherlands; ^4^Department of Pulmonary Disease, University of Groningen, University Medical Center Groningen, Groningen, Netherlands

**Keywords:** checkpoint inhibitors, anti PD-L1 therapy, salivary gland dysfunction, immune related adverse event, cancer treatment, sicca syndrome, hyposalivation

## Abstract

**Background:** Salivary glands (SGs) can be damaged by immune checkpoint inhibitor (ICI) therapy. In patients with ICI-induced SG dysfunction, 60% progress to fulfill classification criteria for primary Sjögren's syndrome (pSS), owing to immune foci in SGs and/or anti-SSA autoantibody positivity. We report the SG tissue analysis of a patient with SG dysfunction after treatment with a programmed death ligand-1 (PD-L1) inhibitor, compared to that of a dry mouth (“sicca”) control and pSS patient.

**Case presentation:** The patient received the PD-L1 inhibitor durvalumab (10 mg/kg, every 2 weeks by intravenous infusion) as adjuvant treatment for stage 3 non-small cell lung carcinoma, following concurrent chemo radiotherapy. At 43 weeks after 21 cycles of Durvalumab, the patient was not capable of producing unstimulated or stimulated parotid gland saliva, and a biopsy was taken. Immunohistochemical analysis showed no classical AQP5^+^ CK7^−^ acinar cell clusters (CK7 marks intercalated ducts, IDs). In contrast, the parenchyma was dominated by hybrid epithelial “structures” with ID-like morphology, containing a mixture of AQP5^+^CK7^−^, AQP5^−^CK7^+^, and AQP5^+^CK7^+^ cells (30 structures/mm^2^). These structures were present at lower frequencies in sicca control (2/mm^2^) and pSS (10/mm^2^) tissue. Hybrid structures contained proliferating (Ki67^+^) cells and senescent (p16^+^) cells. Striated ducts showed no abnormal morphology post PD-L1 treatment, in contrast to pSS tissue. PD-L1 expression was detected in the SG parenchyma following anti-PD-L1 therapy. The SG post-PD-L1 therapy further demonstrated focal lymphocytic sialadentitis, harboring disperse, and focal CD4^+^ T cell-rich infiltrates. CD8^+^ T cells were also present. In this patient, these CD4^+^ and CD8^+^ T cells were observed in-between and inside hybrid structures. CD20^+^ B-cells were infrequently detected following PD-L1 blockade, in contrast to their preponderance in pSS SG tissue.

**Conclusion:** This patient lacked conventional SG acinar cells following anti-PD-L1 therapy and demonstrated presence of hybrid intercalated duct-like structures. Understanding which mechanisms and dynamics underpinning this aberrant parenchyma may be crucial to understand how SG dysfunction post ICI therapy, and potentially other affected organs. Furthermore, although the patient treated with anti-PD-L1 antibody examined here fulfills the criteria for pSS and demonstrated focal lymphocytic sialadentitis, the further histopathological characteristics do not resemble pSS.

## Background

Immune checkpoint inhibitor (ICI) therapy is the engagement of the immune system to kill tumor cells via the blockade of immune system inhibitory checkpoints, mostly employing cytotoxic T-lymphocyte associated protein 4 (CTLA-4), programmed cell death protein-1 (PD-1) and programmed death ligand-1 (PD-L1). ICIs are efficacious in melanoma, lung cancer and head and neck cancer treatment (1). In up to 60% of patients taking ICIs, however, inflammatory diseases such as colitis, pneumonitis, arthritis, inflammatory myopathy, vasculitis, nephritis and sialadentitis, resembling primary Sjögren's syndrome [pSS, including salivary gland (SG) hypofunction] are observed (1–4). “Sicca” syndrome arising from SG hypofunction, including dry mouth, are reported at frequencies of 5% of all patients taking ICIs (1–5).

In a healthy scenario, SG acinar cells produce and secrete saliva, channeled through ducts into the mouth. The vast majority of saliva is produced by the major (parotid, submandibular, and sublingual) SGs, with small contributions from the minor SGs located in the lips and oral cavity. SGs are thought to be maintained by the proliferation and differentiation of tissue resident progenitor cells, largely located in the intercalated and striated ducts (6–9). The observation that ICI-induced hyposalivation cannot be rescued by corticosteroid treatment to dampen inflammation suggests that inflammation is not causing SG hypofunction (3, 10, 11). The SG epithelium is endowed with an ability to receive and transduce inflammatory signals, and actively participate in inflammatory processes, suggesting that a closer examination of the processes following ICI use is crucial to our comprehension of this side-effect (12).

In the case presented here, we compare a parotid SG following prolonged anti-PD-L1 therapy with a sicca control parotid SG, and with a parotid SG from a patient with pSS, to compare the effect of ICI treatment on the SG parenchyma. pSS is an autoimmune disease characterized partly by SG dysfunction, including lymphocytic infiltration, and the presence of anti-SSA autoantibodies, resulting in hyposalivation and oral dryness. SG lymphocytic infiltration in later stages of pSS is predominantly B-cell based, and can also include presence of germinal centers and lymphoepithelial lesions (LELs) (13, 14). An increase in IgG plasma cells in the glands, resulting in <70% IgA plasma cells, further characterizes pSS. Presence of anti-SSA antibodies in blood, and/or a positive focus score (a read-out of SG lymphocytic infiltration extent) plus reduced saliva output leads to classification of our patient as suffering from pSS [ACR-EULAR 2016 criteria (15)]. Studies have suggested that ~60% of patients treated with ICIs and experiencing dry mouth complaints also demonstrate presence of SSA antibodies and/or a positive focus score, designating them technically as suffering from pSS (3, 5, 10, 11). The nature of the lymphocytic infiltration following ICI therapy has been suggested to be CD4^+^ T cell dominated in the minor SGs (3, 5). That of the major SGs and indeed the pathology of the parenchyma, responsible for the majority of saliva production and secretion, has not been documented.

## Case Presentation

The patient (male, 52 years old) received the PD-L1 checkpoint inhibitor durvalumab as an adjuvant treatment for stage 3b non-small cell lung carcinoma of the right upper lobe, following chemo-radiotherapy. Durvalumab was administered intravenously every 2 weeks at a dose of 10 mg/kg, as previously published (16). The patient had a history of psoriasis. The patient completed 1 year, 26 cycles of durvalumab. At cycle 11, he presented clinically with a subjective sensation of dry mouth, “*sicca*” complaints, and was not able to produce any unstimulated or stimulated parotid saliva Additional clinical data is presented in Table 1. Ultrasonography revealed moderate change in SG topography (HOCEVAR score of 14/48), but remained under the threshold score of 15/48 for a positive result (17–19). The Schirmer's test for ocular dryness was positive (4 mm tear fluid /5 min), as opposed to the ocular staining score (OSS), which was negative (0). In order to examine the pathology of the SG, a parotid SG biopsy was performed following our previously published protocol (20).

**Table 1:** Clinical characteristics of patients.

**Characteristics**		**Sicca control**	**pSS**	**Anti-PD-L1**
Sex		Female	Female	Male
Age		68	47	52
Unstimulated whole saliva (ml/min)		ND	0.05	0
Stimulated whole saliva (ml/min)		1.35	0.37	0
Unstimulated parotid saliva (ml/min)	Left	0	0	0
	Right	0	0	0
Stimulated parotid saliva (ml/min)	Left	0	0.09	0
	Right	0.06	0.05	0
Unstimulated submandibular/sublingual saliva (ml/min)	Left + Right	0.02	0.05	0
Stimulated submandibular/sublingual saliva (ml/min)	Left + Right	0.14	0.23	0
Focus score (foci/4mm^2^)		0	2.5	1.0
Lymphoepithelial lesions (LELs)		No	Yes	No
Germinal Center		No	Yes	No
IgG plasma cells		No	No	No
Ultrasound score		–(9/48)	+(26/48)	–(14/48)
Ocular staining score (OSS)		0	3	0
Schirmer's test (mm/5min)		6	0	4
ANA titre		Neg	1:640	1:160
SSA		−	+	+

*ND, no data; ANA, anti-nuclear antibody; HC, healthy control. Ultrasound was scored using the Hocevar et al. scoring system, as validated in Mossel et al., Annals Rheumatic Diseases, 2017 (17). Ocular staining score (OSS) is considered positive for ocular dryness with a score of >3 in at least one eye. Schirmer's test is considered suggestive of ocular dryness with <5 mm tear fluid/5 min in at least one eye. Serum levels of anti-SSA antibodies were assessed with ELISA tests and scored as positive according to standard procedures if more than 10 IU/mL*.

For comparison, parotid SG tissue from a sicca control pSS patient were also analyzed. The sicca control patient demonstrated reduced saliva production, negative HOCEVAR score of 9/48, negative ocular dryness scores and no signs of pSS development (Table 1). The patient with pSS was not capable of producing unstimulated parotid saliva, produced reduced volumes of stimulated parotid saliva (Table 1). The patient with pSS also demonstrated SSA autoantibody positivity, a positive ultrasound score (26/48), and positive test results for ocular dryness (Table 1). All parotid salivary gland biopsies were immunostained as previously reported, with CD45, CD20, CD3, CD4, CD8, Ki67, IgA, IgG, Bcl6, high molecular weight cytokeratins, p16 and PD-L1 (all Ventana) (21). Biopsies were additionally double immunostained for AQP5 and CK7, following antigen retrieval with EDTA buffer (pH = 8) for 15 min. A double staining kit containing secondary antibodies from Thermofisher was used (TL-012-MARH). Primary antibodies of AQP5 (Abcam, clone EPR3747, 1:200) and CK7 (Sigma-Aldrich, clone RCK105, 1:100) were diluted in PBS.

### Lack of Conventional Acinar Cells and Skewing of the Epithelial Compartment Toward Hybrid Intercalated Duct-Like Structures

**Figure 1:**
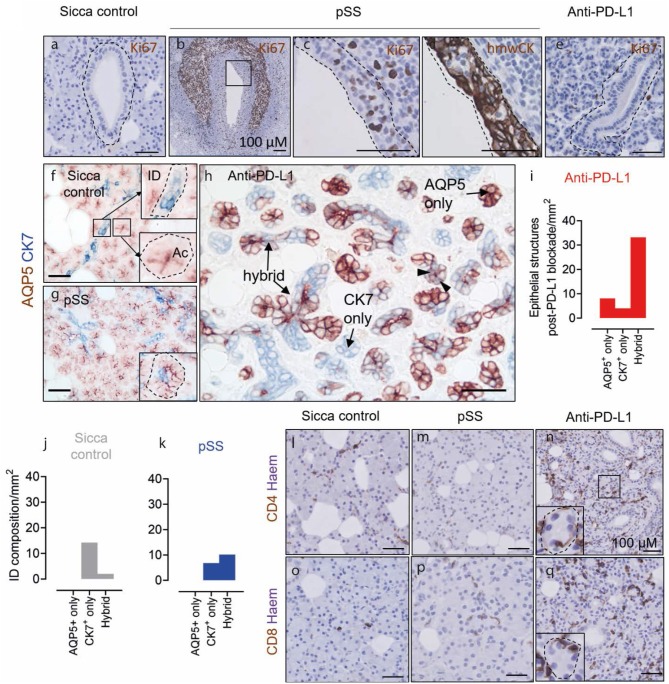
Lack of acinar cells and presence of AQP5^+^ CK7^+^ hybrid epithelial structures in the parotid salivary gland following anti-PD-L1 therapy. **(a,b,c,e)** Ki67 staining in striated ducts of sicca control, pSS and post-anti-PD-L1 therapy tissue. Panels **(c,d)** (immunostained for high molecular weight cytokeratins (hmwCK) to mark SD cells, outlined in dashed line) show high resolution of area in inset in **(b)**. **(f–h)** AQP5 and K7 double immunostaining of sicca control, pSS and post-anti-PD-L1 therapy tissue. Insets in **(f)** shows normal K7 and apical AQP5 staining patterns of intercalated ducts and acinar cell, respectively. Inset in **(g)** show acinar cell clusters in pSS with occasional CK7^+^ cell presence. Block arrows in **(h)** denote AQP5-K7 double-positive cells. **(i)** Quantification of AQP5 and CK7 cell content of epithelial cell structures following anti-PD-L1 blockade. **(j)** Quantification of composition of intercalated duct in sicca control tissue. **(k)** Quantification of composition of intercalated ducts in pSS tissue. **(l,m)** CD4 immunostaining. **(o–q)** CD8 immunostaining. All scale bars represent 50 μm, unless otherwise stated.

In order to examine the effect of anti-PD-L1 therapy on the SG, we examined the striated ducts (SDs), intercalated ducts (IDs) and acinar cells. The SDs of the SG in pSS can undergo invasion by B cells, become proliferative and lead to formation of LELs. The SDs of the sicca control patient demonstrated minimal presence of Ki67^+^ proliferative cells (Figure 1a). Proliferative epithelial cells, defined by immunostaining for Ki67 and high molecular weight cytokeratins (hmwCK) in serial sections, were detected in the pSS tissue (Figures 1b–d). No effect of anti-PD-L1 therapy was apparent on SDs, which displayed a phenotype similar to the sicca control and minimal proliferative cells, marked by Ki67^+^ (Figures 1a,e).

In order to probe how durvalumab effects the acinar and ID cell compartments, we immunostained tissue with AQP5 to mark acinar cells and CK7 to denote IDs. In healthy conventional acinar cells, AQP5 is expressed at the apical cell membrane, and CK7 is not heavily expressed. In sicca control and pSS biopsies, clusters of AQP5^+^CK7^−^ acinar cells with large cytoplasm:nucleus ratios and are easily identifiable (280/mm^2^ sicca control; 170/mm^2^ pSS; Figures 1f,g). AQP5 is localized apically in healthy acinar cell clusters (Figure 1f, inset). In pSS tissue, these conventional acini demonstrated some dysregulation of AQP5 localization, in line with other studies of the minor SGs, but maintained their large cytoplasm:nucleus ratio (22). Acinar clusters in pSS tissue also contained occasional CK7^+^ cells. (Figure 1g inset). In tissue following PD-L1 blockade, AQP5^+^CK7^−^ acinar clusters with large cytoplasm:nucleus ratio and apically located AQP5 were not detectable (Figure 1h). The parenchyma post-PD-L1 blockade was instead dominated by mixture of AQP5^+^CK7^−^, AQP5^−^CK7^+^, and AQP5^+^CK7^+^ cell clusters, whereby AQP5 localization was homogenous throughout the cells and cells maintained a low cytoplasm:nucleus ratio (Figures 1h,i). Interestingly, “hybrid” epithelial structures comprising a mixture of AQP5^+^ and CK7^+^ cells comprised 70% of total epithelial cell clusters (33 structures /mm^2^; Figures 1h,j). Such hybrid epithelial structures were also present in sicca control and pSS tissue, but at much lower frequencies (sicca control 2% of total IDs, 2 IDs/mm^2^; pSS 12%, 10/mm^2^; Figures 1j,k). In sicca control and pSS tissue, CD4^+^ and CD8^+^ T cells were detected sporadically in SG parenchyma (acinar cells plus IDs; Figures 1l,m,o,p). Both T cell subsets were present dispersed at visibly higher frequencies, both in-between and inside epithelial structures following anti-PD-L1 therapy (Figures 1n,q).

### Intercalated Duct-Like Structures Cells Are Proliferative and Potentially Senescent and Following Anti-PD-L1 Therapy

**Figure 2:**
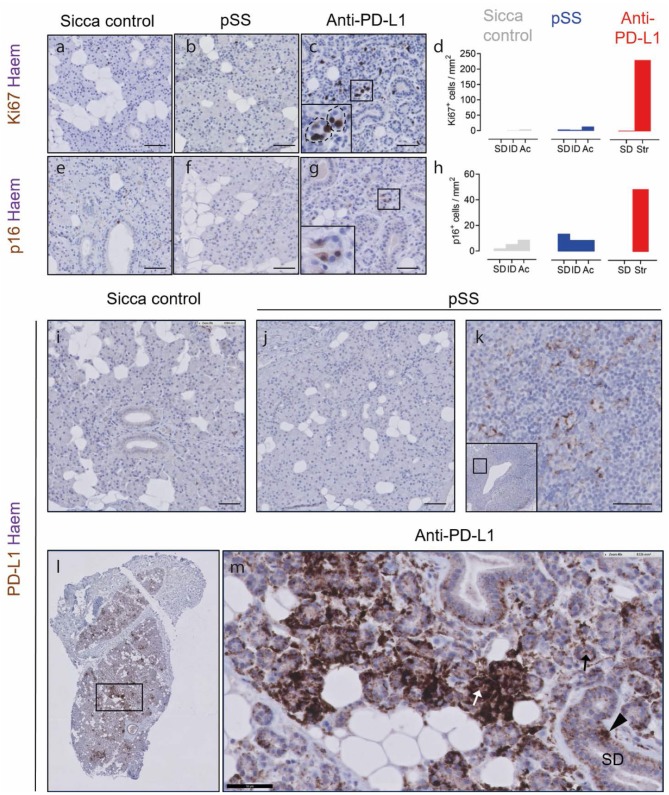
Hybrid epithelial structures following PD-L1 therapy are proliferative, potentially senescent and express PD-L1. **(a–c)** Ki67 immunostaining of sicca control, pSS and post PD-L1 therapy SG tissue. Inset in **(c)** shows high resolution image of boxed area in main panel. **(d)** Quantification of Ki67^+^ epithelial cell types (sicca control and pSS tissue) or hybrid epithelial structures (str; anti-PD-L1 blockade). **(e–g)** Immunostaining of sicca control, pSS and post-PD-L1 blockade tissue for p16. **(h)** Quantification of frequency of p16^+^ cells in epithelial cell types (sicca control and pSS tissue), and hybrid epithelial structures (str; anti-PD-L1 tissue). SDs, striated ducts; ID, intercalated ducts; Ac, acini clusters. **(i)** PD-L1 immunostaining in sicca control. **(j)** PD-L1 immunostaining in pSS tissue. **(k)** PD-L1 immunostaining in germinal center of pSS SG tissue, showing PD-L1^+^ lymphocytes. **(l)** Low resolution microscopy of PD-L1 immunostaining in patient taking durvalumab. **(m)** High resolution image of patient taking durvalumab, showing intensely PD-L1^+^ epithelial structures (white arrow), lightly positive epithelial structures (black arrow) and positive striated duct cells (block arrow). All scale bars represent 50 μM.

In order to probe parenchymal cell dynamics, we immunostained for the senescence marker p16, and Ki67. In the sicca control and pSS parotid SGs, Ki67^+^ proliferating cells were detected at a total frequency of 6/mm^2^ and 24/mm^2^, respectively (Figures 2a,b). Ninety percent of Ki67^+^ cells (equal to 5 cells/mm^2^) were located in the epithelium (SDs, IDs, and acini) in sicca control tissue, and 66% (equal to 16 cells/mm^2^) in pSS tissue (Figure 2d). In tissue following anti PD-L1 therapy, 275 Ki67^+^ cells/mm^2^ were detected, of which 85% (equal to 230 cells /mm^2^) were located within the ID-like epithelial structures (Figures 2c,d). The remaining Ki67^+^ cells were located in SDs (Figures 2c,d).

p16 can be used to denote senescent cells. p16^+^ cells were present at frequencies of 31/mm^2^ in sicca control tissue (Figures 2e,h), and were mostly stromal/non epithelial in nature. We have previously shown that the epithelium of parotid SGs in the autoimmune disease pSS is likely to contain elevated numbers of p16^+^ senescent cells (8). Indeed, p16^+^ cells were detected in pSS tissue at a total frequency of 37/mm^2^ (Figures 2f,h). In pSS tissue, p16^+^ cells were mostly located in striated ducts, in line with our previous studies (13/mm^2^ in SDs, 8/mm^2^ IDs, 9/mm^2^ acinar cells; Figures 2f,h). p16^+^ cells were present in SG tissue following anti-PD-L1 therapy at a frequency of 51/mm^2^, higher than both sicca control and pSS, of which 95% (equal to 48 cells/mm^2^) were located in the ID-like epithelial structures described in Figures 1, 2g,h.

### Intercalated Duct-Like Structures and Striated Ducts Express PD-L1 Following Anti-PD-L1 Therapy

In order to establish if anti-PD-L1 therapy may exert a direct effect on the SG epithelium, we performed immunostaining for PD-L1. In sicca control and pSS tissue, no PD-L1^+^ cells were present in the SG parenchyma (Figures 2i,j). PD-L1^+^ presumptive dendritic cells were detected in the germinal center of pSS tissue (Figure 2k). In tissue following PD-L1 blockade, unconventional epithelial structures were either lightly or intensely PD-L1^+^ (Figures 2l,m). Striated ducts also demonstrated some PD-L1 positivity (Figure 2m).

### Patient Parotid SG Following Prolonged PD-L1 Inhibitor Use Contains Mostly CD4^+^ T Cell Infiltrate and Does Not Resemble pSS Parotid SG Gross Pathology

**Figure 3:**
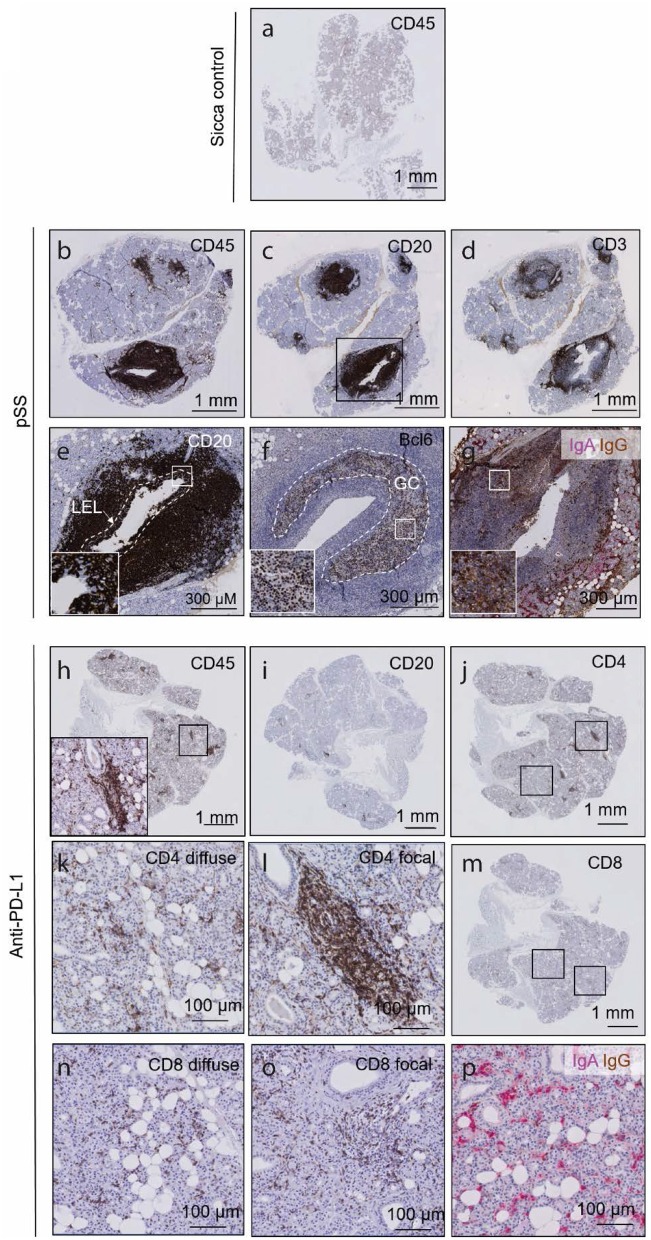
The histology of a parotid salivary gland following prolonged anti PD-L1 therapy contains focal lymphocytic sialadentitis, but does not resemble the parotid gland in pSS. **(a)** CD45 immunohistochemical of sicca control. **(b–d)** CD45, CD20 and CD3 immunohistochemical staining of a parotid salivary gland from a pSS patient. **(e)** High resolution images of a lymphoepithelial lesion, whereby B cells are seen to invade striated ducts. Through as yet unclear mechanisms, both B cells and epithelial cells proliferate, and may represent a precursor stadium to MALT lymphomas. White dashed line demarcates boundaries of original striated duct. Inset box shows CD20^+^ B cells in between CD20^−^ epithelial cells. **(f)** High resolution image of a germinal center, indicated by Bcl-6 positivity. Bcl-6 positive B cells are characteristic of germinal centers. Dashed white line outlines boundaries of germinal center. Inset shows Bcl-6-positive nuclei of B cells. **(g)** Double immunohistochemical staining for IgA and IgG classes of antibodies. IgA represents the isotype normally found in mucosal tissues such as the SG (red label). Where >80% of labeled cells are IgG positive (brown), class switching is assumed to have occurred, such as the present example. **(h–n)** CD45, CD20, CD8, and CD4 immunohistochemical staining of a parotid salivary gland from patient following prolonged anti-PD-L1 therapy. Insets in **(h)** shows high resolution of boxed area. **(k)** High resolution image of diffuse CD8 immunostaining. **(l)** High resolution image of focally organized CD8 immunostaining. **(n)** High resolution image of focally organized CD4 immunostaining. **(o)** High resolution image of focal CD8 immunostaining. **(p)** IgA IgG double immunostaining following prolonged anti-PD-L1 therapy, showing majority of pink, IgA class antibodies.

In order to compare SG histopathological findings (i.e., nature of sialadentitis, presence of LELs, IgG plasma cells, and germinal centers) in a patient with sicca following PD-L1 blockade to that observed in pSS, we performed immunohistochemical staining for infiltrating lymphoid cells. Sicca control parotid SGs show dispersed, scarce CD45^+^ cell presence, no focal CD45^+^ cell presence, no focal lymphocytic sialadentitis, no LELs, germinal centers or IgG plasma cell presence (Figure 3a). These features were all present in the parotid biopsy of the pSS patient examined (Figures 3b–g; clinical details in Table 1). CD45^+^ leukocytes were present throughout the biopsy post PD-L1 blockade, both periductally and dispersed between the epithelial cells of the parenchyma (Figure 3h). The infiltrate was composed of a majority of CD4^+^ T cells, in addition to considerable presence of CD8^+^ T cells, and a minor contribution to total infiltrate from CD20^+^ B cells (Figures 3i–o). CD4^+^ and CD8^+^ lymphocytes present were both diffusely dispersed through the tissue and focally clustered around the striated ducts, in a similar manner to pSS (“focal sialadentitis”; Figures 3k,l,n,o). No LELs, germinal centers or IgG secreting plasma cells were observed in this biopsy (Figures 1p, 3, Table 1). A focus score of 1.0 was calculated for the anti-PD-L1 treated patient (the focus score of the pSS patient was 2.5). Together with the lack of saliva production, this focus score would lead to classification of this patient post-PD-L1 therapy as suffering from pSS.

## Discussion and Conclusions

In this case study, we examine parotid SG morphology of a patient with sicca complaints following anti-PD-L1 therapy, with particular attention for the epithelium. The most striking observation was the lack of cells responsible for producing saliva, namely conventional acinar cell clusters with apically located AQP5 expression and high cytoplasm:nucleus ratio. Intercalated duct-like epithelial structures composed of a mixture of AQP5^+^CK7^−^, AQP5^−^CK7^+^, and AQP5^+^CK7^+^ cells dominated the parenchyma instead, whereby AQP5 was mislocalized. Considering the lack of parotid SG function by this patient, these structures are presumably not capable of saliva production. This appears to be in contrast with the phenotype of the minor SGs observed post-ICI use, whereby conventional acinar cells are still present, suggesting striking differences in the reactionary abilities between major and minor SGs (3, 5). The phenotype is also dissimilar to parotid SG dysfunction induced by radiation, where saliva production is lost immediately following radiation (as opposed to around 70 days post ICI commencement), and where tissue will demonstrate both fibrosis and acinar cell cluster loss (23).

A predominance of CD4^+^ T cell lymphocytic infiltration in the parotid SG post-PD-L1 therapy was observed, although CD8^+^ T cell presence was also considerable. CD20^+^ B-cells were nearly absent. This is in agreement with literature examining minor SGs, where mostly CD4^+^ infiltrate was observed (3, 5). CD4^+^ and CD8^+^ T cells were found both focally around but not inside SDs, in addition to dispersed throughout the tissue. Both CD4^+^ and CD8^+^ T cells also infiltrated the ID-like epithelial structures post PD-L1 blockade. Interestingly, CD8^+^ T cells and CD4^+^ T cells were also found sporadically in acinar cell clusters of SG tissue from sicca control and pSS patients, albeit at lower frequency. These data may suggest that CD8^+^ cell presence in the SG parenchyma is detrimental to SG/acinar function, considering neither sicca control or pSS patient produced normal amounts of saliva (24).

The phenotype of the SG described in the current case study raises questions about the sequence of events occurring in the SG following anti-PD-L1 therapy, and their effect on acinar cells. PD-L1 expression was greater in the SG parenchyma after anti-PD-L1 blockade, compared to sicca and pSS control tissues. Which stimuli originally triggers this increased PD-L1 expression, and any effect thereafter on SG function of epithelial PD-L1 interaction with PD-L1 blocking therapeutics such as durvalumab remains to be clarified. Existing studies have documented a relationship between PD-L1 expression and interferon-γ levels (IFNγ), whereby IFNγ has been shown to increase PD-L1 expression, and PD-L1 expression to be protective against interferon-γ induced toxicity (25, 26). CD4^+^ and CD8^+^ T cells recruited to the SG may be responsible for the gross alterations in parotid SG dynamics observed, although which particular CD4^+^ or CD8^+^ subsets are responsible will require phenotyping of the infiltrate. Proliferative (Ki67^+^) and potentially senescent (p16^+^) epithelial cells were detected in this SG post PD-L1 therapy. The ability to proliferate implies that a SG resident progenitor cell population may have been affected by anti-PD-L1 therapy, directly or indirectly. The observed skewing toward unconventional epithelial structures may represent an attempt by progenitor cells to restore acinar cell balance, which may have not reached completion.

Sixty percent of patients experiencing lack of saliva production following checkpoint inhibitor use will progress to fulfill classification criteria for pSS. Recent studies suggest that small proportion (7 or 15%, in recent reports) of these patients display anti-SSA positivity, with the remaining fulfilling classification criteria owing to positive focus scores (3, 5). SGs affected by pSS, at least in its advanced stages, are notable for their preponderance of CD20^+^ B cells, in addition to germinal center, LELs, and IgG producing plasma cell presence. These features were not present in the SG examined here following anti-PD-L1 therapy, in line with recent reports examining the minor SGs. Thus although foci are present, the nature of the infiltrate and the pathology observed are not consistent with the classical SG of a pSS patient (3, 5). The parotid SG examined in this case does not resemble that in pSS, histopathologically, despite the presence of anti-SSA in the serum.

In conclusion, our observations suggest that epithelial tissues such as the SG can react dramatically to anti-PD-L1 therapy. Our data supports a recent observation by Warner et al., who reported that hyposalivation following ICI therapy cannot be resolved with anti-inflammatory corticosteroid use. These data imply that the presence of infiltration is not the reason for lack of SG function observed. According to our data, the SG epithelium may be grossly dysregulated following PD-L1 blockade (3). Whether ICI therapy targeting other receptors (anti-CTLA4; anti-PD-1) also results in loss of acinar structures remains to be shown. Exhaustive additional studies will be necessary to comprehend fully the mechanisms behind PD-L1 blockade induced SG dysfunction, to understand how to affect tumor cell removal using ICIs with minimal epithelial organ dysfunction.

## Ethics Statement

Salivary gland biopsy was performed as part of a diagnostic work up procedure for primary Sjögren's syndrome, under Institutional Review Board approval numbers METc2013.066, METc 2018-558. Patients gave informed consent for tissue use. Written informed consent was obtained from the individuals for the publication of any potentially identifiable images or data included in this article.

## Author Contributions

SP: conception, design, analysis, and interpretation. BV and NB: acquisition and interpretation. XW and TH: acquisition. FS: interpretation. AV and HB: interpretation and draft writing. FK: interpretation, draft writing, and design. All authors read and approved the final manuscript.

### Conflict of Interest

The authors declare that the research was conducted in the absence of any commercial or financial relationships that could be construed as a potential conflict of interest.
